# Assessing early left ventricular remodeling in pediatric hypertension: A study using transthoracic echocardiography combined with two‐dimensional speckle tracking echocardiography in an immature rabbit model

**DOI:** 10.1002/pdi3.92

**Published:** 2024-07-07

**Authors:** Lingxin Feng, Xu Zhu, Xiaojuan Ji, Huiru Zhu, Tingting Ran, Haiyan Yang

**Affiliations:** ^1^ Department of Ultrasound Children's Hospital of Chongqing Medical University National Clinical Research Center for Child Health and Disorders Ministry of Education Key Laboratory of Child Development and Disorders Chongqing China; ^2^ Chongqing Key Laboratory of Pediatrics Chongqing China; ^3^ State Key Laboratory of Ultrasound in Medicine and Engineering Chongqing Medical University Chongqing China; ^4^ Department of Ultrasound The Third Affiliated Hospital of Chongqing Medical University Chongqing China; ^5^ Department of Ultrasound Chongqing General Hospital Chongqing University Chongqing China; ^6^ Department of Ultrasound Chongqing Health Center for Women and Children Women and Children's Hospital of Chongqing Medical University Chongqing China

**Keywords:** 2D‐STE, echocardiography, left ventricular remodeling, myocardial fibrosis, pediatric hypertension

## Abstract

This study explored the value of routine transthoracic echocardiography (TTE) combined with two‐dimensional speckle tracking echocardiography (2D‐STE) for the early evaluation of left ventricular remodeling in the hypertensive immature rabbit model. Twenty‐seven New Zealand white rabbits were divided into Group A (sham‐operated group), Group B (mild group), and Group C (severe group), with 9 rabbits per group. The hypertension model was constructed using the “two kidneys one clip” method. Changes in left ventricular function and the degree of left ventricular wall thickening were observed by TTE at 1, 4, and 8 weeks after modeling. The global longitudinal strain (GLS‐AVG, GLS‐A4C, GLS‐A2C, and GLS‐LAX) of the left ventricle (LV) and the longitudinal strain (LS) of the 18 segments of left ventricular myocardium were analyzed using 2D‐STE. Concurrently, LV myocardial tissue was sampled for HE staining and Masson staining. Receiver operating characteristic (ROC) curves were plotted to evaluate the accuracy of 2D‐STE parameters in predicting myocardial fibrosis. The model group exhibited varying degrees of left ventricular remodeling. GLS‐A4C, GLS‐A2C, GLS‐LAX, and GLS‐AVG in the model group increased at 1 week after modeling (*P* < 0.01), with LS abnormalities concentrated in the apical segments. GLS‐AVG showed a significant positive correlation with both IVSd and CVF (*P* < 0.01). The area under the curve (AUC) values of GLS‐AVG, GLS‐A4C, GLS‐A2C, and GLS‐LAX were 0.850, 0.827, 0.839, and 0.800, respectively. This study demonstrates the promise of TTE combined with 2D‐STE for the early and comprehensive evaluation of left ventricular myocardial damage in hypertensive children in the clinical setting.

## Graphical abstract

Different degrees of hypertension immature rabbit model showed varying degrees of left ventricular hypertrophy and myocardial fibrosis. The combination of TTE and 2D‐STE can provide early and comprehensive evaluation of left ventricular remodeling in the model, including changes in left ventricular structure and function, and corresponding pathological features.

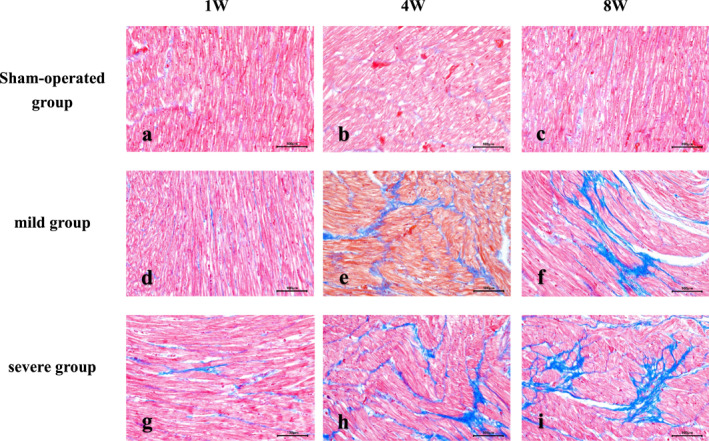

## 1 INTRODUCTION

There is a rising trend of hypertension among younger individuals, and the global prevalence of pediatric hypertension is approximately 4.0%.^1^ Pediatric hypertension can primarily stem from familial genetics or excessive obesity and may also be secondary to vascular stenosis, kidney disease, endocrine disorders, and other factors.^2^ The persistent elevation of blood pressure subjects cardiomyocytes to long‐term overload, rendering them more susceptible to damage. This can lead to left ventricular remodeling changes, such as left ventricular dilatation, myocardial hypertrophy, myocardial fibrosis, and other alterations.^3^ In severe cases, these changes can result in heart failure and arrhythmias, closely correlating with the occurrence of cardiovascular events and a poor prognosis.^4^ Timely detection of changes in cardiac function in pediatric hypertension and early intervention are valuable for improving prognosis.^5^ Current studies on pediatric hypertension have mainly focused on observing hemodynamic changes,^6^ with structural and pathological changes in the heart being rarely reported. In this study, we employed Transthoracic Echocardiography (TTE) combined with Two‐Dimensional Speckle Tracking Echocardiography (2D‐STE) to conduct a preliminary exploration of changes in left ventricular structure, left heart function, and corresponding pathological alterations in an immature rabbit model of hypertension.

## 2 MATERIALS AND METHODS

### 2.1 Establishment of the hypertension immature rabbit model

Twenty‐seven male New Zealand white rabbits, aged 2 months, with body weight (BW) ranging from 2.0 to 2.5 kg, were randomly divided into three groups of nine rabbits each: Group A (sham‐operated group), Group B (mild group), and Group C (severe group). The rabbits were administered with 3% pentobarbital sodium (30 mg/kg) intravenously via the ear vein for anesthesia. Following anesthesia, the abdomen was opened in the right lateral position. After isolating the intestines, the left renal artery was bluntly detached from the middle part for about 1 cm, and the abdomen was then closed in Group A. U‐shaped silver clips with internal diameters of 0.7 and 0.2 mm were applied approximately 1.5 cm from the left renal hilum in Group B and Group C, respectively. The abdomen was closed after surgery. Postoperatively, 400,000 units of penicillin were injected intramuscularly for three consecutive days, and the animals were routinely fed for 8 weeks. This experiment was approved by the Animal Ethics Committee of Chongqing Medical University Animal Experiment Center.

### 2.2 Echocardiography

#### 2.2.1 Instrument

We utilized the Philips EPIQ 7C color Doppler ultrasonic diagnostic instrument equipped with an S9‐2 phased array probe, operating at a frequency range of 1–9 MHz and a detection depth of 3–5 cm. The frame rate for 2D‐STE was set at > 50 frames/sec. Additionally, the equipment was equipped with QLAB ultrasonic image processing workstation. Image analysis was performed by Image J software (National Institutes of Health).

#### 2.2.2 Observation indicators

At 1, 4, and 8 weeks after modeling, animals in each group were shaved on the chest and limbs and intraperitoneally injected with 3% pentobarbital sodium (30 mg/kg). They were then connected to ECG electrodes and placed supine on the examination bed or in the lateral position if necessary. Cardiac ultrasound scanning and mapping were performed by the same senior sonographer.

TTE indicators: Relevant indicators were measured by M‐mode ultrasound in the parasternal long‐axis view of the LV and included Interventricular septal thickness at diastole (IVSd), left ventricular posterior wall thickness at end‐diastole (LVPWd), left ventricular posterior wall thickness at end‐systole (LVPWs), left ventricular internal diameter at end‐diastole (LVIDd), left ventricular internal diameter at end‐systole (LVIDs), end‐diastolic volume (EDV), and end‐systolic volume (ESV). Left ventricular ejection fraction (LVEF) and left ventricular fractional shortening (LVFS) were measured using the biplane Simpson method.

Strain analysis by 2D‐STE: After stabilizing the heart rate, depth, gain, and other parameters were adjusted to maintain consistency and ensure clear imaging. Images of the left ventricular four‐chamber view, two‐chamber view, and three‐chamber view for 6–8 consecutive cardiac cycles were stored in a two‐dimensional ultrasound mode. Then, opened AutoStrain LV software package (Philips EPIQ Release 5.0) and the endocardium of the LV were outlined by the same operator. The tracing line was fitted as closely as possible to the endocardium, and the average global longitudinal strain (GLS‐AVG) of LV, the global longitudinal strain of each view (GLS‐A4C, GLS‐A2C, GLS‐LAX), and the longitudinal strain (LS) of 18 segments were calculated by the software. The LV was divided into 18 segments according to the American Society of Echocardiography standards.^7^

### 2.3 Pathological indicators

Three experimental animals in each group were randomly selected for euthanasia by an overdose of anesthesia after echocardiography. The left ventricular tissue was excised, fixed in a 4% paraformaldehyde solution, embedded in paraffin, and sectioned. Hematoxylin and eosin (HE) staining was used to observe the morphology of cardiomyocytes, and Masson staining was employed to assess the degree of myocardial fibrosis. From each Masson‐stained section, six fields of view with the strongest positive expression were selected and imported into Image J image analysis software to calculate the collagen volume fraction (CVF) and derive the average value. CVF was calculated using the formula: CVF = area of collagen fiber expression in the field of view/area of tissue in the field of view.

### 2.4 Statistical analysis

Statistical analysis was performed using SPSS Stat 26 software (SPSS Inc). All data were presented as mean ± standard deviation. Kolmogorov–Smirnov tests were conducted to assess data normality. For variables that followed a normal distribution, the one‐way analysis of variance was employed to compare differences in mean values, and Pearson correlation analysis was performed to examine correlations. For variables that did not follow a normal distribution, the Wilcoxon rank‐sum test was employed to compare differences in mean values, and Spearman correlation analysis was performed to examine correlations. A *p*‐value of less than 0.05 (*P* < 0.05) was considered statistically significant.

## 3 RESULTS

All animals in this study exhibited no infections or deaths for 8 weeks after surgery.

### 3.1 Characteristics of changes in routine TTE indicators

At 1 week after modeling, compared with Group A, there were no statistically significant differences in the indicators in Group B (*P* > 0.05). However, in Group C, IVSd was increased (*P* < 0.05), and LVIDd, EDV, and ESV were decreased (*P* < 0.05).

At 4 weeks after modeling, compared with Group A, LVEF and LVFS were decreased in Group B and Group C (*P* < 0.05). IVSd, LVIDd, LVPWs, and EDV were increased (*P* < 0.01), and LVIDs were increased in Group C (*P* < 0.05). There were no statistically significant differences in all TTE indicators between Group B and Group C.

At 8 weeks after modeling, compared with Group A, LVEF and LVFS were decreased in Group B and Group C (*P* < 0.05), and the remaining TTE indicators were increased (*P* < 0.05). Statistically significant differences were observed between Group B and Group C in IVSd, LVPWs, EDV, and LVFS (*P* < 0.05) (Refer to Table 1).

**TABLE 1:** Routine TTE indicators of hypertension models.

Groups indicators	Group A (*n* = 9)			Group B (*n* = 9)			Group C (*n* = 9)		
	1 W	4 W	8W	1 W	4 W	8 W	1 W	4 W	8 W
IVSd (cm)	0.23 ± 0.01	0.24 ± 0.06	0.26 ± 0.01	0.24 ± 0.01	0.33 ± 0.02^b^	0.36 ± 0.03^b^	0.28 ± 0.03^ac^	0.35 ± 0.01^b^	0.41 ± 0.02^bc^
LVIDd (cm)	1.28 ± 0.06	1.33 ± 0.03	1.34 ± 0.08	1.25 ± 0.07	1.42 ± 0.05^a^	1.45 ± 0.45^b^	1.18 ± 0.04^a^	1.44 ± 0.12^b^	1.47 ± 0.05^b^
LVIDs (cm)	0.82 ± 0.08	0.83 ± 0.06	0.85 ± 0.09	0.82 ± 0.03	0.87 ± 0.08	0.95 ± 0.13^b^	0.81 ± 0.06	0.91 ± 0.04^a^	1.01 ± 0.04^b^
LVPWd (cm)	0.22 ± 0.02	0.21 ± 0.02	0.22 ± 0.01	0.23 ± 0.01	0.24 ± 0.02	0.28 ± 0.04^b^	0.24 ± 0.03	0.25 ± 0.01	0.31 ± 0.02^b^
LVPWs (cm)	0.25 ± 0.01	0.26 ± 0.05	0.28 ± 0.02	0.28 ± 0.03	0.30 ± 0.02^a^	0.35 ± 0.06^b^	0.30 ± 0.03	0.31 ± 0.02^a^	0.38 ± 0.04^bc^
EDV (ml)	4.31 ± 0.31	4.51 ± 0.39	4.53 ± 0.71	4.22 ± 0.24	4.71 ± 0.16^a^	4.83 ± 0.85^b^	4.16 ± 0.67^a^	5.13 ± 1.04^b^	5.26 ± 1.86^bd^
ESV (ml)	1.55 ± 1.53	1.52 ± 0.11	1.54 ± 0.24	1.53 ± 0.06	1.54 ± 0.33	1.59 ± 0.54^a^	1.32 ± 0.13^ac^	1.57 ± 0.21	1.61 ± 0.21^a^
LVEF (%)	63.25 ± 0.12	61.60 ± 0.05	67.65 ± 0.08	56.20 ± 0.02	53.45 ± 0.15^b^	47.80 ± 0.03^b^	58.75 ± 0.45	52.10 ± 1.22^b^	44.64 ± 0.55^b^
LVFS (%)	36.83 ± 1.39	34.91 ± 2.02	32.89 ± 2.46	33.75 ± 0.14	31.61 ± 1.15^a^	28.55 ± 0.06^a^	32.11 ± 0.15	29.54 ± 0.55^b^	25.75 ± 1.12^bc^

*Note*: Data were given as mean ± standard deviation. Compared with Group A, ^a^ *P* < 0.05. ^b^ *P* < 0.01. Compared with Group B, ^c^ *P* < 0.05, ^d^ *P* < 0.01.

Abbreviations: EDV, end‐diastolic volume; ESV, end‐systolic volume; IVSd, interventricular septal thickness at diastole; LVEF, left ventricular ejection fractions; LVFS, left ventricular fractional shortening; LVIDd, left ventricular internal diameter at end‐diastole; LVIDs, left ventricular internal diameter at end‐systole; LVPWd, left ventricular posterior wall thickness at end‐diastole; LVPWs, left ventricular posterior wall thickness at end‐systole.

### 3.2 Characteristics of changes in 2D‐STE indicators

#### 3.2.1 GLS of the LV

At 1 week after modeling, GLS‐LAX was higher in Group C than in Group B (*P* < 0.05). At 4 weeks after modeling, GLS‐A2C, and GLS‐AVG were higher in Group C than in Group B (*P* < 0.05). At 8 weeks after modeling, GLS‐A2C, GLS‐LAX, and GLS‐AVG were higher in Group C than in Group B (*P* < 0.05).

At weeks 1, 4, and 8 after modeling, GLS‐A4C, GLS‐A2C, GLS‐LAX, and GLS‐AVG were significantly higher in Group B and Group C compared with Group A (*P* < 0.01) (Refer to Table 2).

**TABLE 2:** GLS of hypertension models.

Groups indicators	Group A (*n* = 9)			Group B (*n* = 9)			Group C (*n* = 9)		
	1W	4 W	8W	1 W	4W	8 W	1W	4 W	8W
GLS‐A4C (%)	−20.93 ± 1.08	−23.67 ± 1.10	−23.23 ± 2.51	−17.70 ± 0.52^b^	−14.47 ± 1.08^b^	−12.07 ± 2.78^b^	−18.67 ± 0.93^a^	−12.00 ± 1.25^b^	−10.07 ± 0.55^b^
GLS‐A2C (%)	−24.60 ± 1.56	−21.27 ± 1.81	−22.07 ± 0.67	−16.60 ± 1.50^b^	−16.34 ± 2.60^a^	−13.97 ± 2.98^b^	−17.43 ± 1.34^b^	−10.50 ± 4.20^ad^	−9.60 ± 1.47^bd^
GLS‐LAX (%)	−21.40 ± 1.65	−22.87 ± 1.60	−21.17 ± 1.87	−18.23 ± 1.01^ac^	−13.47 ± 0.72^b^	−12.17 ± 0.76^b^	−15.87 ± 2.30^bc^	−13.83 ± 2.34^b^	−9.10 ± 0.44^bd^
GLS‐AVG (%)	−22.30 ± 0.75	−22.60 ± 1.39	−22.17 ± 1.50	−17.50 ± 0.53^b^	−14.73 ± 1.05^b^	−13.43 ± 1.02^b^	−17.37 ± 0.78^b^	−12.10 ± 2.26^bc^	−9.60 ± 1.50^bd^

*Note*: Data were given as mean ± standard deviation. Compared with Group A, ^a^ *P* < 0.05. ^b^ *P* < 0.01. Compared with Group B, ^c^ *P* < 0.05, ^d^ *P* < 0.01.

Abbreviations: GLS‐A2C, global longitudinal strain at the two‐chamber view; GLS‐A4C, global longitudinal strain at the four‐chamber view; GLS‐AVG, average global longitudinal strain; GLS‐LAX, global longitudinal strain at the long axis view.

**FIGURE 1:**
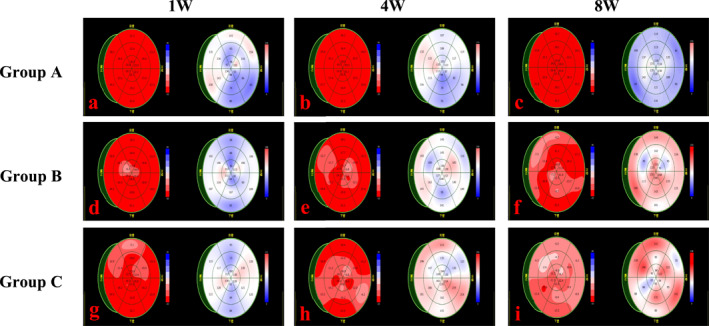
(a–i) The left image is the GLS bull's eye plot, right image is the peak time bull's eye plot. (a–c) In Group A, the GLS bull's eye plots were uniformly red, and the peak time bull's eye plots were predominantly blue, suggesting that the left ventricular myocardial systolic function was normal. (d–f) In Group B, the color of GLS bull's eye plots became lighter from the apical region, and the peak time bull's eye plots appeared red in the corresponding segments, indicating abnormal systolic function from the apical segments. The abnormal area increased gradually, suggesting extensive myocardial damage. (g–i) In Group C, GLS and peak time bull's eye plots showed a large area of abnormality that progressively worsened over time.

The comparative analysis of GLS bull's eye plots in the hypertension immature rabbit model revealed that the abnormal apical segment systolic function occurred 1 week after modeling in Group B. The extent of myocardial damage gradually increased with modeling time. In contrast, a large area of myocardial damage appeared 1 week after modeling in Group C (Refer to Figure 1).

#### 3.2.2 LS of left ventricular segments

At 1 week after modeling, compared with Group A, there were 6 segments increased in Group B (*P* < 0.05), primarily in the anterolateral and inferoseptal segments, and 8 segments increased in Group C (*P* < 0.05), mainly in the anteroseptal and inferoseptal segments.

At 4 weeks after modeling, compared with Group A, there were 10 segments increased in Group B (*P* < 0.05), mainly in the inferolateral and inferoseptal segments, and 14 segments increased in Group C (*P* < 0.05), primarily in the inferior, inferolateral, anteroseptal, and inferoseptal segments.

**FIGURE 2:**
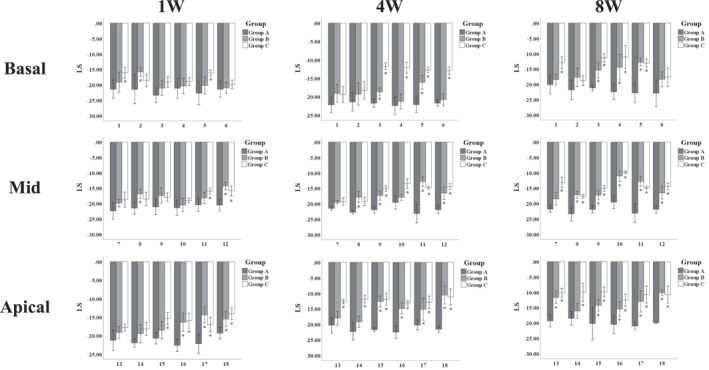
LS of left ventricular segments. 1. basal anterior; 2. basal anterolateral; 3. basal inferolateral; 4. basal inferior; 5. basal inferoseptal; 6. basal anteroseptal; 7. mid anterior; 8. mid anterolateral; 9. mid inferolateral; 10. mid inferior; 11. mid inferoseptal; 12. mid anteroseptal; 13. apical anterior; 14. apical anterolateral; 15. apical inferolateral; 16. apical inferior; 17. apical inferoseptal; 18. apical anteroseptal. At 1 week after modeling, compared with Group A, the increased segments in Group B were 2, 8, 12, 16, 17, and 18; in Group C, the increased segments were 1, 5, 11, 12, 15, 16, 17, and 18. At 4 weeks after modeling, compared with Group A, the increased segments in Group B were 3, 5, 8, 9, 11, 12, 15, 16, 17, and 18; in Group C, all segments were increased except 1, 2, 7, and 8. At 8 weeks after modeling, compared with Group A, in Group B, all segments were increased except 1, 2, 6, 7, and 14; in Group C, all segments were increased except 2 and 6. Compared with Group A, *P < 0.05.

At 8 weeks after modeling, compared with Group A, 13 segments were increased in Group B (*P* < 0.05), and 16 segments were increased in Group C (*P* < 0.05), indicating extensive abnormalities of multisegmental LS (Refer to Figure 2).

### 3.3 Pathological indicators

#### 3.3.1 HE staining

**FIGURE 3:**
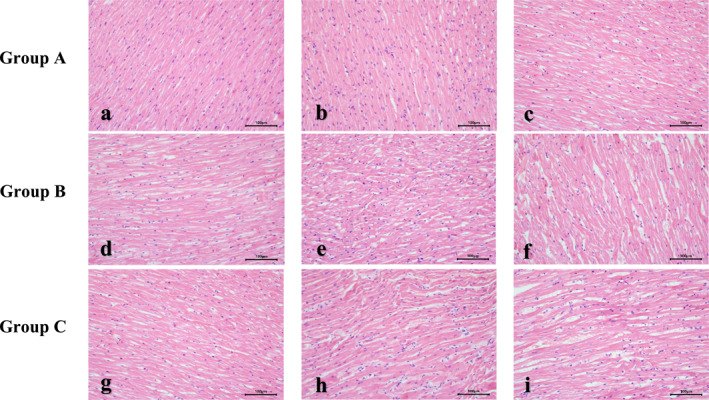
(a–i) HE staining of the left ventricular myocardium of hypertension models (×200).

HE staining was utilized to observe the morphology and structure of left ventricular cardiomyocytes: In Group A, the morphology of cardiomyocytes was normal and densely arranged, with a clearer structure. At 1 week after modeling, the morphology of cardiomyocytes in Group B and Group C was acceptable, and left ventricular remodeling was not obvious. However, at 4 weeks, cardiomyocytes showed an increase in size, disorganization of arrangement, compression, and destruction of normal morphology, with a gradually widening intercellular space. The degree of damage to cardiomyocytes further deepened at 8 weeks (Refer to Figure 3).

#### 3.3.2 Masson staining

**FIGURE 4:**
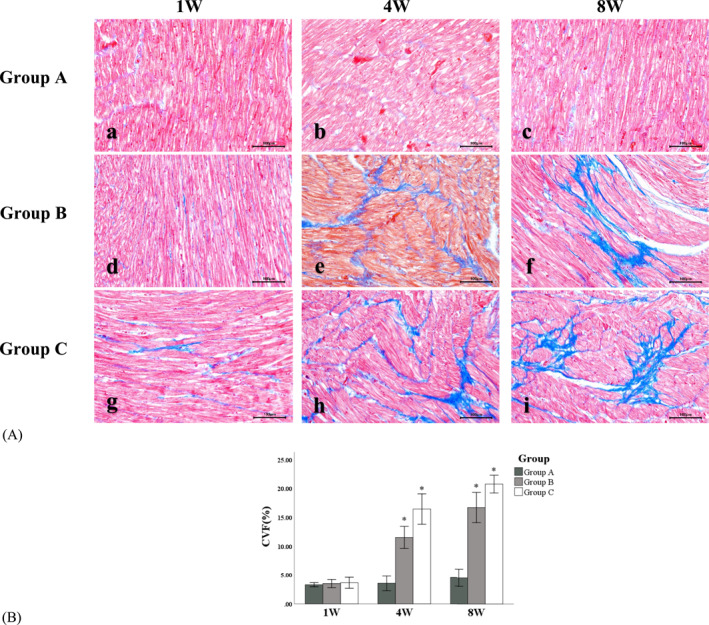
(A) (a–i) Masson staining of the left ventricular myocardium of hypertension models (×200). (B) CVF of hypertension models. Compared with Group A, *P < 0.05.

Masson staining was employed to assess the degree of myocardial fibrosis: In Group A, there were no apparent fibrotic changes in the cardiomyocytes, with only a small amount of blue collagen fibers expressed in the intercellular space. However, Group B and Group C exhibited more pronounced interstitial fibrosis at 4 weeks after modeling, characterized by the increased deposition of collagen fibers in the intercellular space. This expression progressively increased over time, with a gradual increase in the CVF (Refer to Figure 4).

### 3.4 Correlation analysis of GLS‐AVG with IVSd and CVF

**FIGURE 5:**
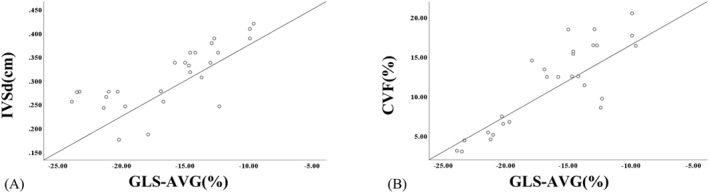
(A) Scatterplot of correlation between GLS‐AVG and IVSd. (B) Scatterplot of correlation between GLS‐AVG and CVF.

The correlation coefficient between GLS‐AVG and IVSd is 0.742 (*P* < 0.01), indicating a strong positive correlation. Similarly, the correlation coefficient between GLS‐AVG and CVF is 0.851 (*P* < 0.01), suggesting a strong positive correlation as well (Refer to Figure 5).

### 3.5 Predictive value of 2D‐STE GLS parameters for myocardial fibrosis

**FIGURE 6:**
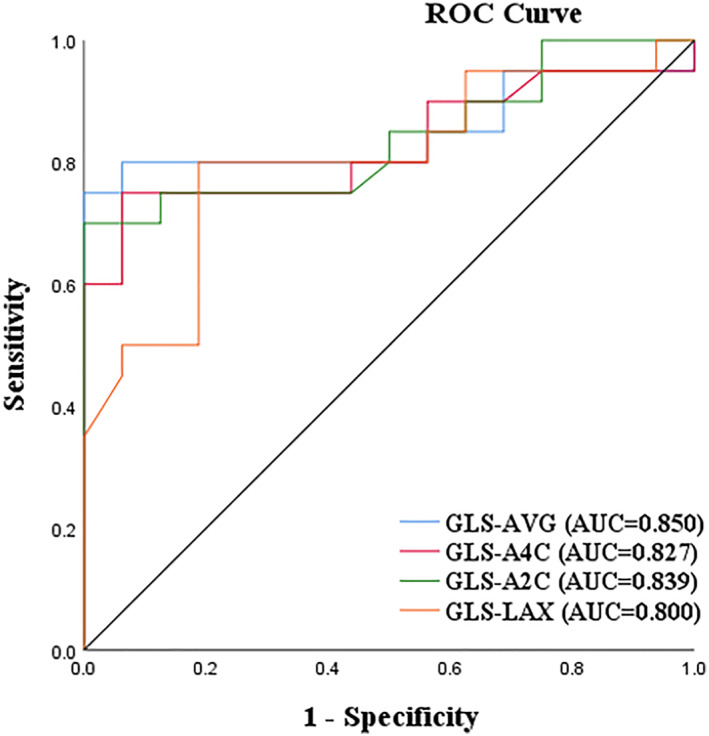
ROC curve of the predictive value of 2D‐STE GLS parameters for myocardial fibrosis.

The Area Under the Curve (AUC) values of GLS‐AVG, GLS‐A4C, GLS‐A2C, and GLS‐LAX were 0.850, 0.827, 0.839, and 0.800, respectively. The sensitivity and specificity of GLS > −13.9% for predicting myocardial fibrosis were 0.75 and 0.71, respectively (Refer to Figure 6).

## 4 DISCUSSION

Previous studies have demonstrated that employing silver clips with varying internal diameters to narrow renal arteries in the “two kidneys one clip” method can induce different degrees of hypertension‐related changes in blood pressure.^8^, ^9^, ^10^ In this study, two types of silver clips, 0.7 and 0.2 mm, were utilized to induce hypertension in immature rabbits. This allowed for the dynamic observation of structural effects and pathological changes in juvenile left ventricular myocardium under different degrees of hypertension.

Routine TTE follow‐up revealed a transient decrease in LVIDd in the model group compared to the sham‐operated group 1 week after modeling, followed by a progressive increase. This increase was significantly higher than that of the sham‐operated group at 4 weeks after modeling. The authors attribute this phenomenon to compensatory inward thickening of the left ventricular wall in the early stages of modeling.^11^ Over time, cardiomyocytes became further damaged and decompensated, leading to continued thickening of the left ventricular wall with left ventricular cavity dilatation. The observed thickening of IVSd at 4 weeks after modeling suggests the early onset of pathological myocardial hypertrophy due to sustained blood pressure elevation. Although LVPWd also exhibited a thickening trend, it only reached statistical significance at 8 weeks after modeling, indicating typical asymmetric cardiac hypertrophic changes in the LV of the immature rabbit hypertension model constructed using the “two kidneys one clip” method.^12^

Increased left ventricular afterload in hypertension predominantly affects subendocardial longitudinally aligned myocardium. Therefore, the abnormal GLS of the LV reflects early left ventricular motor dysfunction.^13^ Previous studies have shown that abnormalities in GLS precede left ventricular diastolic dysfunction and hypertrophy in adult hypertensive patients.^14^ Similarly, in this study, GLS‐A4C, GLS‐A2C, GLS‐LAX, and GLS‐AVG increased at 1 week after modeling, despite LVEF measurements remaining within the normal range. This suggests that GLS indicators also reflect left ventricular remodeling in hypertensive juvenile myocardium at an early stage, consistent with previous findings. LVEF, which reflects global changes in LV geometry, may not accurately detect abnormalities in myocardial segmental systolic function and filling pressures.^15^ Additionally, abnormalities in myocardial systolic function are often subclinical in the early stages of hypertension, with myocardial segmental systolic function impairments preceding changes in LVEF.^16^ 2D‐STE is more sensitive in the early assessment of the impaired left ventricular systolic function by directly tracking myocardial segment motion.^17^, ^18^ At 4 and 8 weeks after modeling, the model group exhibited progressive increases in GLS, myocardial thickness, and CVF of LV, indicating ongoing left ventricular remodeling worsened left ventricular systolic dysfunction, and reduced strain capacity over time. Notably, GLS‐LAX was higher in Group C than in Group B at 1 week after modeling, suggesting potential differences in left ventricular systolic dysfunction even when GLS‐AVG values are similar.

Myocardial segmental LS abnormalities in the model group appeared predominantly in apical segments 1 week after modeling. Over time, the number of abnormal LS segments gradually increased at 4 and 8 weeks after modeling, with the most significant increase observed from week 1 to week 4. This indicates the progression of hypertension‐induced left ventricular remodeling from partial to complete involvement of the LV, underscoring the importance of early detection of cardiac dysfunction and timely intervention to protect cardiac function and improve the prognosis of hypertensive children. Abnormalities in left ventricular wall segmental motion were most pronounced in the anteroseptal and inferoseptal segments, consistent with changes in interventricular septum myocardial thickness, highlighting the corresponding relationship between left ventricular myocardial segmental hypertrophy and motion dysfunction in hypertensive children.^19^

Hypertension‐induced changes such as myocardial ischemia and hypertrophy lead to metabolic disorders in cardiomyocytes, activating myocardial fibroblasts differentiation to myofibroblasts and resulting in myocardial fibrosis.^20^ This excessive collagen fiber deposition in the extracellular matrix correlates directly with the degree of left ventricular dysfunction.^21^ In this study, the degree of myocardial fibrosis, quantified by calculating CVF, showed left ventricular myocardial fibrosis at 4 weeks after modeling, primarily manifested as interstitial fibrosis. CVF increased progressively over time, indicating more severe fibrosis with longer disease duration. Correlation analysis revealed significant positive correlations between GLS‐AVG and IVSd, as well as CVF, suggesting that 2D‐STE can sensitively reflect myocardial hypertrophy and fibrosis degree, enabling the preliminary assessment of myocardial pathological changes in hypertensive children.

The predictive value of 2D‐STE parameters for myocardial fibrosis was assessed by ROC analysis. The AUC values of GLS‐AVG, GLS‐A4C, GLS‐A2C, and GLS‐LAX ranged from 0.80 to 0.85, indicating a good predictive value. GLS‐AVG had the highest AUC of 0.850, with a sensitivity of 75% and specificity of 71% for predicting myocardial fibrosis using a cut‐off value of GLS‐AVG > −13.9%, which was close to previous studies.^22^, ^23^ This suggests that 2D‐STE has a significant application value in assessing myocardial fibrosis. In clinical practice, when 2D‐STE is applied to evaluate cardiac function in hypertensive children, GLS‐AVG > −13.9% is highly suggestive of myocardial fibrosis, necessitating early intervention to prevent adverse cardiovascular events.

## 5 CONCLUSION

In summary, this study highlights the potential of 2D‐STE in the early detection and assessment of left ventricular remodeling in hypertensive children. By rapidly obtaining strain values of global LV and myocardium segments, 2D‐STE can detect early changes in local left ventricular systolic function, assess the degree of hypertension‐induced myocardial hypertrophy, and even evaluate myocardial fibrosis. This provides valuable information for the clinical evaluation of left ventricular systolic function and pathological progression in pediatric hypertension. Additionally, these findings can aid in the development of diagnostic and therapeutic protocols, enabling early intervention in left ventricular remodeling and ultimately improving the prognosis of hypertensive children.

## AUTHOR CONTRIBUTIONS

Dr. Xiaojuan Ji contributed to the conception and investigation of the study. Huiru Zhu and Lingxin Feng constructed the hypertension models. Xu Zhu and Tingting Ran performed echocardiography on the models. Haiyan Yang and Lingxin Feng assisted in data collation and statistical analysis. Dr. Xiaojuan Ji and Lingxin Feng drafted the manuscript. All authors approved the final manuscript as submitted and agreed to be accountable for all aspects of the work.

## CONFLICT OF INTEREST STATEMENT

The authors declare that the research have no conflicts of interest.

## ETHICS STATEMENT

The study was approved by the Institutional Animal Care and Use of Chongqing Medical University (Approval number: IACUC‐CQMU‐2024‐0194).

## Data Availability

The data that support the findings of this study are available from the corresponding author upon reasonable request.
